# Comparative Transcriptome Analysis of Waterlogging-Sensitive and Tolerant Zombi Pea (*Vigna vexillata*) Reveals Energy Conservation and Root Plasticity Controlling Waterlogging Tolerance

**DOI:** 10.3390/plants8080264

**Published:** 2019-08-02

**Authors:** Pimprapai Butsayawarapat, Piyada Juntawong, Ornusa Khamsuk, Prakit Somta

**Affiliations:** 1 Department of Genetics, Faculty of Science, Kasetsart University, Bangkok 10900, Thailand; 2 Center for Advanced Studies in Tropical Natural Resources, National Research University-Kasetsart University, Bangkok 10900, Thailand; 3 Omics Center for Agriculture, Bioresources, Food and Health, Kasetsart University (OmiKU), Bangkok 10900, Thailand; 4 Department of Botany, Faculty of Science, Kasetsart University, Bangkok 10900, Thailand; 5 Department of Agronomy, Faculty of Agriculture at Kamphaeng Saen, Kasetsart University, Nakhon Pathom 73140, Thailand

**Keywords:** *De novo* transcriptome, lateral root, legume, *Vigna vexillata*, waterlogging

## Abstract

*Vigna vexillata* (zombi pea) is an underutilized legume crop considered to be a potential gene source in breeding for abiotic stress tolerance. This study focuses on the molecular characterization of mechanisms controlling waterlogging tolerance using two zombi pea varieties with contrasting waterlogging tolerance. Morphological examination revealed that in contrast to the sensitive variety, the tolerant variety was able to grow, maintain chlorophyll, form lateral roots, and develop aerenchyma in hypocotyl and taproots under waterlogging. To find the mechanism controlling waterlogging tolerance in zombi pea, comparative transcriptome analysis was performed using roots subjected to short-term waterlogging. Functional analysis indicated that glycolysis and fermentative genes were strongly upregulated in the sensitive variety, but not in the tolerant one. In contrast, the genes involved in auxin-regulated lateral root initiation and formation were expressed only in the tolerant variety. In addition, cell wall modification, aquaporin, and peroxidase genes were highly induced in the tolerant variety under waterlogging. Our findings suggest that energy management and root plasticity play important roles in mitigating the impact of waterlogging in zombi pea. The basic knowledge obtained from this study can be used in the molecular breeding of waterlogging-tolerant legume crops in the future.

## 1. Introduction

Flooding is one of the most significant problems facing global agriculture today. It can be categorized as waterlogging, i.e., when the height of the water column covers only the root-zone, or as submergence, when the aerial plant tissues are fully covered [1]. Waterlogging generally affects dryland crops rather than submergence, since soil can easily become waterlogged due to poor drainage after intensive and/or extensive rainfall or irrigation. Waterlogging creates low oxygen environments in the root, due to the limited diffusion of oxygen and other gases under water. This results in ATP shortage from the inhibition of oxidative phosphorylation, and long-term waterlogging results in stomatal closure, leading to impaired root hydraulic conductivity and reduced photosynthesis and nutrient and water uptake in the plants [2].

The characterization of the molecular mechanisms for submergence tolerance has been extensively studied in model plants. Functional characterization of group VII of ethylene response factor (ERF) genes revealed their functional role as critical players regulating submergence tolerance in rice and *Arabidopsis* [3,4,5]. In rice, natural genetic variations of group VII ERFs determine the escape strategy through stem elongation in the deepwater rice and the quiescence strategy through the restriction of shoot elongation in the lowland rice [6,7]. In another monocot model, *Brachypodium distachyon*, transcriptomic analysis of submergence-tolerant and sensitive natural genetic variations revealed the oxidative stress pathway as a significant tolerance factor [8]. Most of the submergence and low oxygen studies in model plants provide some basic understanding; however, these studies were frequently conducted in a hypoxic environment under complete darkness, which cannot imitate the impact observed in plant response to waterlogging [2].

The legume family (*Fabaceae*) is one of the most important food crops for human nutritional needs. However, molecular characterization of the mechanisms controlling waterlogging tolerance in the legume family is uneven. Most existing studies on the molecular basis of waterlogging tolerance in legumse were focused on soybean. A key component associated with waterlogging stress in soybean is an energy crisis in root-zone resulting from low oxygen conditions, with the meristem showing particular susceptibility. Waterlogging-tolerant soybean varieties were found to develop more aerenchyma and promote more root growth than the sensitive varieties under waterlogging stress [9,10,11,12]. The natural genetic diversity of soybean has been used to find molecular mechanisms that are differentially expressed in tolerant versus sensitive varieties [13,14,15,16,17]. Recently, a major quantitative trait locus (QTL), *qWT_Gm_03*, controlling root plasticity under waterlogging was identified in soybean and proposed to be involved in the auxin pathways regulating secondary root development and root plasticity [17]. In other legume species, higher root porosity and the ability to form lateral roots was also correlated with waterlogging tolerance, as observed in the waterlogging-tolerant legume of the genus *Trifolium* [18], pea (*Pisum sativum*) [19], and lentil (*Lens culinaris*) [19].

The genus *Vigna* is a particularly important legume crop, comprising more than 1000 species and distributed in extensive and diverse areas of Africa, America, Australia, and Asia [20,21]. Domesticated *Vigna* species including cowpea (*V. unguiculata*), zombi pea (*V. vexillata*), Bambara groundnut (*V. subterranean*), mungbean (*V. radiata*), azuki bean (*V. angularis*), rice bean (*V. umbellata*), black gram (*V. mungo*), moth bean (*V. aconitifolia*), and créole bean (*V. reflexo-pilosa*) are grown mainly for dry seeds by small farmers in several cropping systems of tropical and sub-tropical regions [22,23]. Most of the domesticated *Vigna* species are particularly sensitive to waterlogging, resulting in poor seed quality and significant yield reduction. In the case of mungbean, waterlogging at the vegetative stage results in decreased leaf area, growth rate, root growth, photosynthesis rate, chlorophyll and carotenoid contents, flowering rate, pod setting, yield, and altered dry matter partitioning [24]. In contrast to soybean, little is known about the molecular mechanisms of waterlogging tolerance in the genus *Vigna*. Therefore, to improve *Vigna* waterlogging tolerance, mechanisms of waterlogging tolerance must be understood. It has been proposed that stress-resistant plant species closely related to the crop of interest could be used for the molecular analysis of the stress adaptation mechanisms [25]. Thus, de novo transcriptome analysis and gene expression profiling can be used to provide a basic understanding of the molecular response controlling waterlogging adaptation of the non-model *Vigna* crops.

*Vigna vexillata* (common name: zombi pea) is an underutilized legume crop that can be found in diverse areas of Africa, America, Australia, and Asia [26]. It is cultivated for edible storage roots and seeds. Zombi pea is a highly heterogeneous legume species [21]. Previous research has found that some varieties of zombi pea adapt well to environmental stresses including infertile soil [27], alkaline soil [28], drought [29], and waterlogging [30]. Therefore, zombi pea is considered to be a potential gene source in breeding for tolerance to abiotic stresses.

In this work, we investigated the changes in anatomy, morphology, and molecular responses to waterlogging with the assistance of RNA-sequencing (RNA-seq) of the waterlogged roots of two zombi pea varieties with contrasting waterlogging-tolerant phenotypes. We hypothesized that the natural genetic diversity of the zombi pea would allow us to find the molecular mechanism of waterlogging tolerance in the genus *Vigna*.

## 2. Results and Discussion

### 2.1. Anatomical and Morphological Changes of Zombi Pea Varieties Subjected to Waterlogging Stress

**Figure 1:**
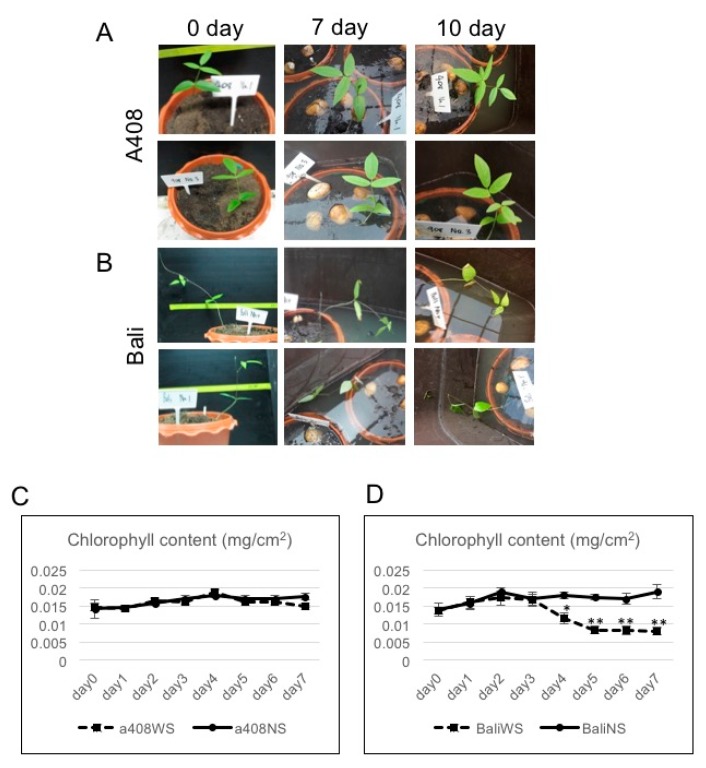
Contrasting waterlogging tolerance in “A408” and “Bali” varieties. Representative 15-day-old zombi pea seedlings subjected to 0, 7, and 10 days of waterlogging. (**A**) “A408”. (**B**) “Bali”. Leaf chlorophyll measurement (*n* = 12 plants) under no stress (NS) and waterlogging (WS). (**C**) “A408”. (**D**) “Bali”. * *p* < 0.05, ** *p* < 0.01 (*t*-test).

Two zombi pea varieties, the waterlogging-tolerant “A408” and the waterlogging-sensitive “Bali”, were selected based on the contrasting phenotype in response to waterlogging. “A408”, a native pasture on the verge of a swamp, is highly waterlogging-tolerant [30]. “Bali” is an Asian cultivated zombi pea found in Bali, Indonesia [21]. The contrasting phenotype of these natural varieties was initially tested by growing them in pot soils and waterlogging for 30 days (data not shown). In this study, we applied waterlogging at the seedlings stage (15 day-old). Under long-term waterlogging stress (WS), “A408” was able to maintain growth based on the visual examination (Figure 1A). On the other hand, “Bali” displayed stunted growth of its phenotype under WS (Figure 1B). Analysis of leaf chlorophyll content demonstrated that under non-stress (NS), “A408” maintained its chlorophyll content between 0.014 mg/cm^2^ at day zero and 0.017 mg/cm^2^ at day seven (Figure 1C). Similarly, “Bali” chlorophyll contents ranged between 0.014 mg/cm^2^ at day zero and 0.018 mg/cm^2^ at day seven under NS (Figure 1D). Further, WS did not affect “A408” chlorophyll content (Figure 1C). In contrast, the reduction of “Bali” chlorophyll content was observed between day four (0.01 mg/cm^2^) and day seven (0.008 mg/cm^2^) of WS (Figure 1D), suggesting that WS resulted in a decline in “Bali” leaf photosynthesis. The decrease in leaf chlorophyll content under WS was also observed in other WS-sensitive legume varieties [19,31].

**Figure 2:**
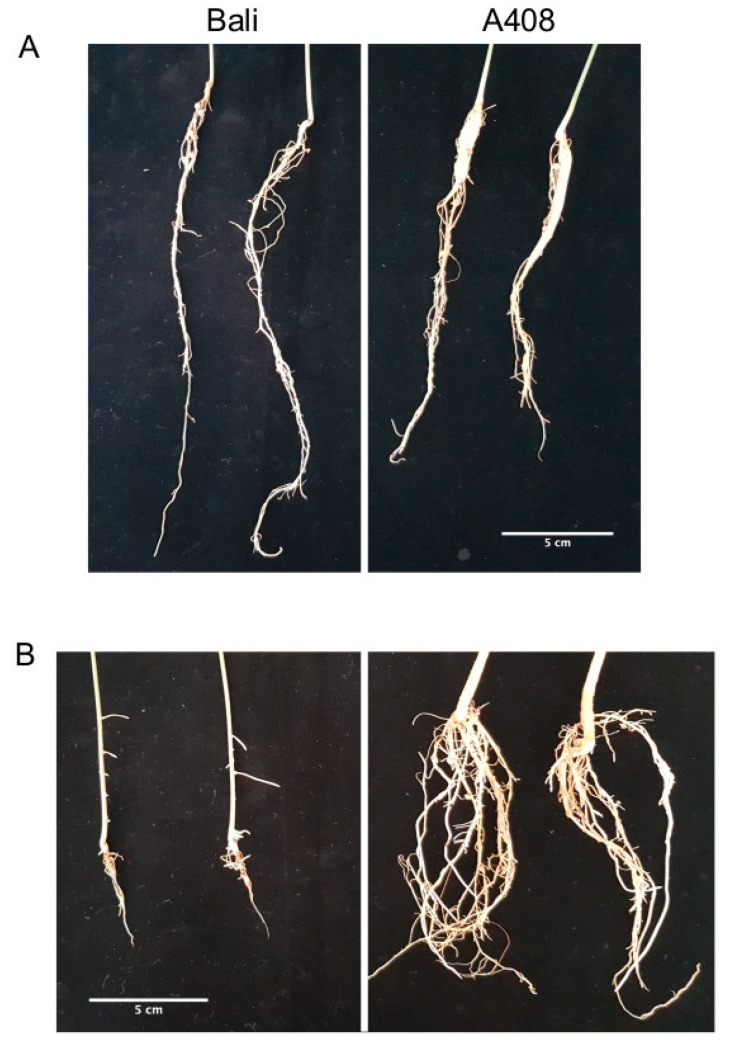
Changes of zombi pea root architecture under WS. (**A**) Roots of control plants kept for 7 days under NS. (**B**). Roots of 7-day WS plants.

**Figure 3:**
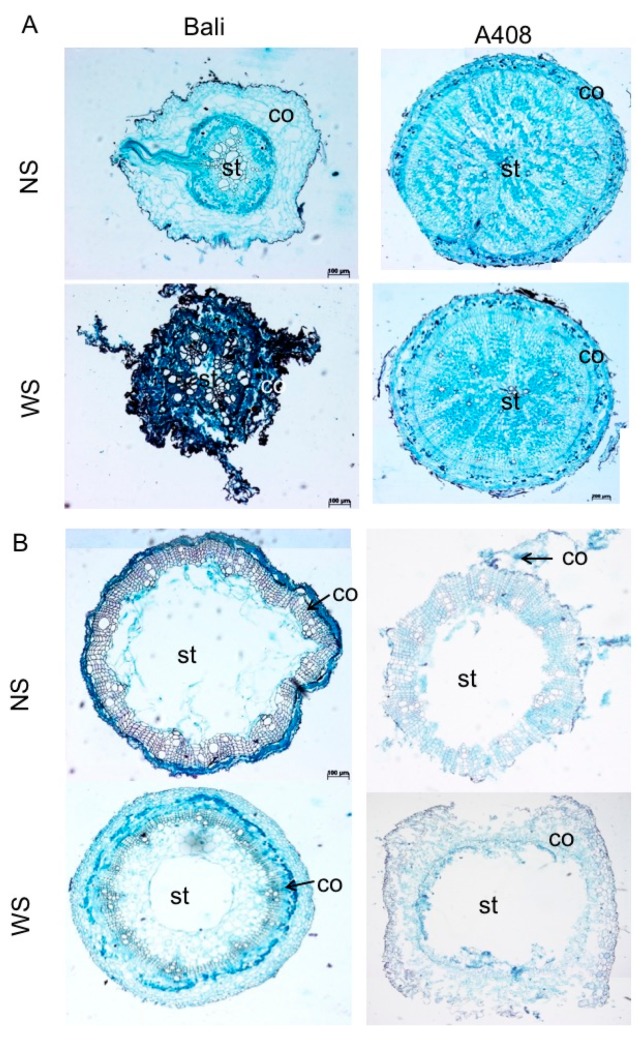
Waterlogging induces aerenchyma and extra-cellular airspace in hypocotyls and roots of “A408”. Cross-section of “A408” and “Bali” (**A**) taproot and (**B**) hypocotyl. co = cortex. st = stele.

Root architecture and plasticity play a vital role in the adaptation of plants to WS [32]. Therefore, we analyzed for WS-induced root anatomical and morphological changes in zombi pea and found that WS caused damage and significantly suppressed the root growth of “Bali” (Figure 2A,B). On the other hand, WS promoted basal stem thickening and lateral root production in “A408” (Figure 2A,B). Lateral roots are all roots that emerge from main roots, and are a major determinant of root architecture, which is essential for the efficient uptake of water and nutrients [33]. To determine the taproot anatomy, roots at the same age were sectioned at almost the same position (Figure 3A). In the cutting area, we observed the secondary root growth in “A408” but the primary root growth in “Bali”. We randomly cut “Bali” roots in different root zones, but only the primary root growth was observed. Based on “A408” anatomy, “A408” taproots functions as storage roots. Therefore, the cortical region of A408 taproot was smaller than that of “Bali”. Furthermore, we observed numerous starch grains in the parenchyma of “A408” root steles. In addition, WS resulted in the formation of aerenchyma in taproots and hypocotyls of “A408” variety (Figure 3A,B, respectively). In contrast, WS caused severe tissue damage in taproot of “Bali”, as observed by dark precipitation of Fast Green dye, and no aerenchyma was observed in WS hypocotyls of “Bali” (Figure 3A,B). The formation of aerenchyma was responsible for increasing internal oxygen diffusion from the aerial parts to the waterlogged roots, which allowed the underground roots to maintain aerobic respiration [34]. Our results correlated with the three previous studies in other legume crops. First, in waterlogging-tolerant legumes of the genus *Trifolium*, higher root porosity and the ability to form lateral roots contributed to waterlogging tolerance [18]. Second, in waterlogging-tolerant pea and lentil, WS increased both the main and lateral root porosity compared to the NS due to the formation of aerenchyma [19]. Lastly, a soybean locus, *qWT_Gm_03*, enhanced waterlogging tolerance through controlling secondary root growth in a waterlogging-tolerant cultivar [17]. Since lateral root formation was induced by WS in “A408”, but root growth was arrested in WS “Bali” (Figure 2A,B), these results suggest that the plasticity in lateral root development under WS could be an important determinant for waterlogging tolerance in zombi pea.

### 2.2. De Novo Transcriptome Analysis

To examine the molecular mechanisms controlling waterlogging tolerance in zombi pea, we performed de novo transcriptome analysis by RNA-seq using WS and NS root samples derived from both “A408” and “Bali” varieties. Twenty-two to twenty-six million reads were obtained for each RNA-seq library (Table S1). To construct a reference transcriptome for each variety, the RNA-seq reads from four libraries (two biological replicates per each treatment) were combined and subjected to de novo transcriptome assembly by Trinity program. The transcriptome assembly yielded 74,658 genes consist of 154,405 transcripts with an average transcript length of 1263 bp (N50 = 2134 bp) and GC content of 39.70%, and 80,279 genes consisting of 173,848 transcripts with an average transcript length of 1230 bp (N50 = 2087 bp) and GC content of 39.74% for “A408” and “Bali”, respectively (Files S1 and S2; Table S1). The de novo transcriptome assembly statistics were similar between the two varieties.

To perform functional characterization of the de novo assembled transcriptomes, the candidate open reading frames of each transcript (>100 amino acids; 94,801 and 106,142 protein-coding transcripts from “A408” and “Bali”, respectively) were annotated using BLASTP to plant UniprotPK database to obtain the associated gene ontology (GO) terms and assigned to functional bins by Mercator pipeline (Table S2). Transcript homologs among “A408”, “Bali”, and *Arabidopsis* were identified by the OrthoVenn2 web tool. Transcript expression, as represented by count per million (CPM) expression values can be found in Table S3.

### 2.3. Differential Gene Expression, Functional Enrichment, and Comparative Transcriptome Analyses

**Figure 4:**
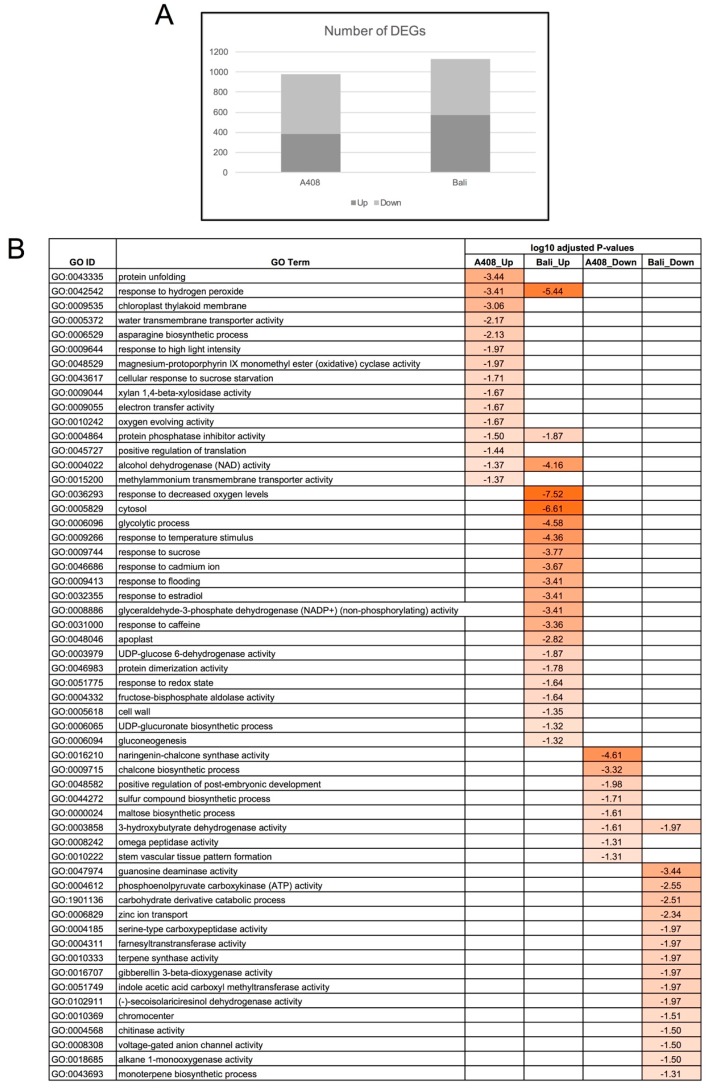
Waterlogging altered root transcriptomes of “A408” and “Bali”. (**A**) The number of upregulated and downregulated differentially-expressed genes (DEGs) from roots of “A408” and “Bali” in response to WS. (**B**) Enrichment of GO terms from upregulated and downregulated DEGs from roots of “A408” and “Bali” in response to WS.

For differential gene expression analysis, reads were mapped back to the assembled transcriptome. The majority of reads (96–97%) from each RNA-seq library could be mapped to the reference transcriptome (Table S1), suggesting the reliability of our transcriptome data. The number of reads aligned back to each transcript was acquired for differential gene expression analysis. Transcriptome analysis identified 982 differentially-expressed genes (DEGs) and 1133 DEGs with significant changes in gene expression evaluated by the false discovery rate (FDR) <0.05 from “A408” and “Bali”, respectively (Figure 4A; Table S3). For “Bali”, 51% and 49% of DEGs were upregulated and downregulated by WS (Figure 4A; Table S3). On the other hand, a higher percentage of DEGs (61%) were downregulated compared to the percentage of upregulated DEGs (39%) in “A408” (Figure 4A, Table S3).

Using a list of core hypoxia-induced genes in *Arabidopsis* [35], we were able to identify 31 core hypoxia homolog clusters among “A408”, “Bali”, and *Arabidopsis* (Table S3). Of these, four homolog clusters, including *sucrose synthase* (cluster 56), *alcohol dehydrogenase* (cluster 3967), *similar to RCD one 5* (*SRO5*; cluster 4428), and *wound-responsive family protein* (cluster 8884), were induced in both “A408” and “Bali” (Table S3). *Non-symbiotic hemoglobin 1* (cluster 13294) was induced only in “A408”. In contrast, *1-aminocyclopropane-1-carboxylate oxidase 1* (*ACC oxidase 1*; cluster 15158), *haloacid dehalogenase-like hydrolase (HAD) superfamily protein* (cluster 6574), and *LOB domain-containing protein 41* (*LBD41*, cluster 9883) were specifically induced in “Bali”.

**Figure 5:**
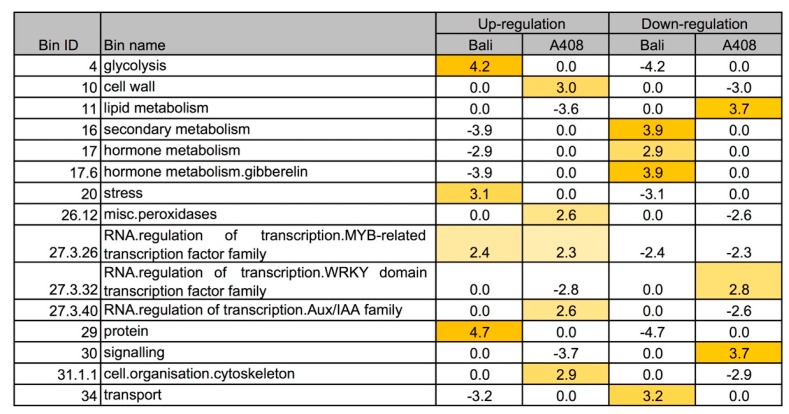
Comparative transcriptome response for selected functional categories to WS in roots of “A408” and “Bali”. Over-representation analysis of the DEGs (FDR < 0.05). The statistical analysis of overrepresented functional categories was performed using Fisher method. Z-scores indicate over/under representation. (Number indicates z-score; Yellow indicates over-representation). Data used to generate this figure can be found in Table S4.

We took two contemporary approaches to identify differentially-expressed molecular mechanisms controlling waterlogging tolerance; GO enrichment analysis of co-expressed genes (Figure 4B) and comparative transcriptome analysis (Figure 5). To obtain a global picture of transcriptome adjustment in response to WS, GO enrichment analysis was carried out. The top five GO terms of upregulated DEGs of “A408” were enriched for protein unfolding, response to hydrogen peroxide, chloroplast thylakoid membrane, water transmembrane transporter activity, and asparagine biosynthetic process (Figure 4B). On the other hand, the top five GO terms of upregulated DEGs of “Bali” were enriched in response to decreased oxygen levels, cytosol, response to hydrogen peroxide, glycolytic process, and response to temperature stimulus (Figure 4B). Response to hydrogen peroxide, protein phosphatase inhibitor activity, and alcohol dehydrogenase (NAD) activity were the common GO terms that were identified from the upregulated DEGs of both “A408” and “Bali” (Figure 4B). The top five downregulated DEGs of “A408” were enriched for naringenin-chalcone synthase activity, chalcone biosynthetic process, positive regulation of post-embryonic development, sulfur compound biosynthetic process, and maltose biosynthetic process (Figure 4B). In “Bali”, the top five downregulated DEGs were enriched in guanosine deaminase activity, phosphoenol pyruvate carboxykinase activity, carbohydrate derivative catabolic process, zinc ion transport, serine-type carboxypeptidase activity, farnesyltranstransferase activity, terpene synthase activity, gibberellin 3-beta-dioxygenase activity, indole acetic acid carboxyl (IAA) methyltransferase activity, (-)-secoisolariciresinol dehydrogenase activity, and 3-hydroxybutyrate dehydrogenase activity (Figure 4B).

To compare the changes in WS transcriptome in the two zombi pea varieties with contrasting WS responses, comparative transcriptome analysis was analyzed by over-representation analysis (ORA) using Fisher’s exact test with a cut-off of two. The results from the ORA analysis demonstrated that glycolysis, stress, MYB-related transcription factor family, and protein functional bins were overrepresented in the upregulated DEGs of “Bali” (Figure 5; Table S4). In contrast, the upregulated DEGs of “A408” were overrepresented with cell wall, peroxidase, MYB-related transcription factor family, AUX/IAA transcription factor family, and cytoskeleton functional bins (Figure 5; Table S4). The downregulated DEGs of “Bali” were overrepresented with secondary metabolism, hormone metabolism (including gibberellin), and transport functional bins (Figure 5; Table S4). For “A408”, the downregulated DEGs were overrepresented with lipid metabolism, WRKY transcription factor family, and signaling functional bins (Figure 5; Table S4). The results from the GO enrichment and the ORA analyses point out that differential regulation of the genes encoding for energy production pathways, hormones, RNA-regulation by AUX/IAA family, cell wall modification, water transmembrane transporters, and peroxidase enzymes could contribute to waterlogging tolerance in zombi pea.

### 2.4. Waterlogging Resulted in Stronger Induction of Glycolysis and Fermentative Genes in “Bali” than in “A408”

**Figure 6:**
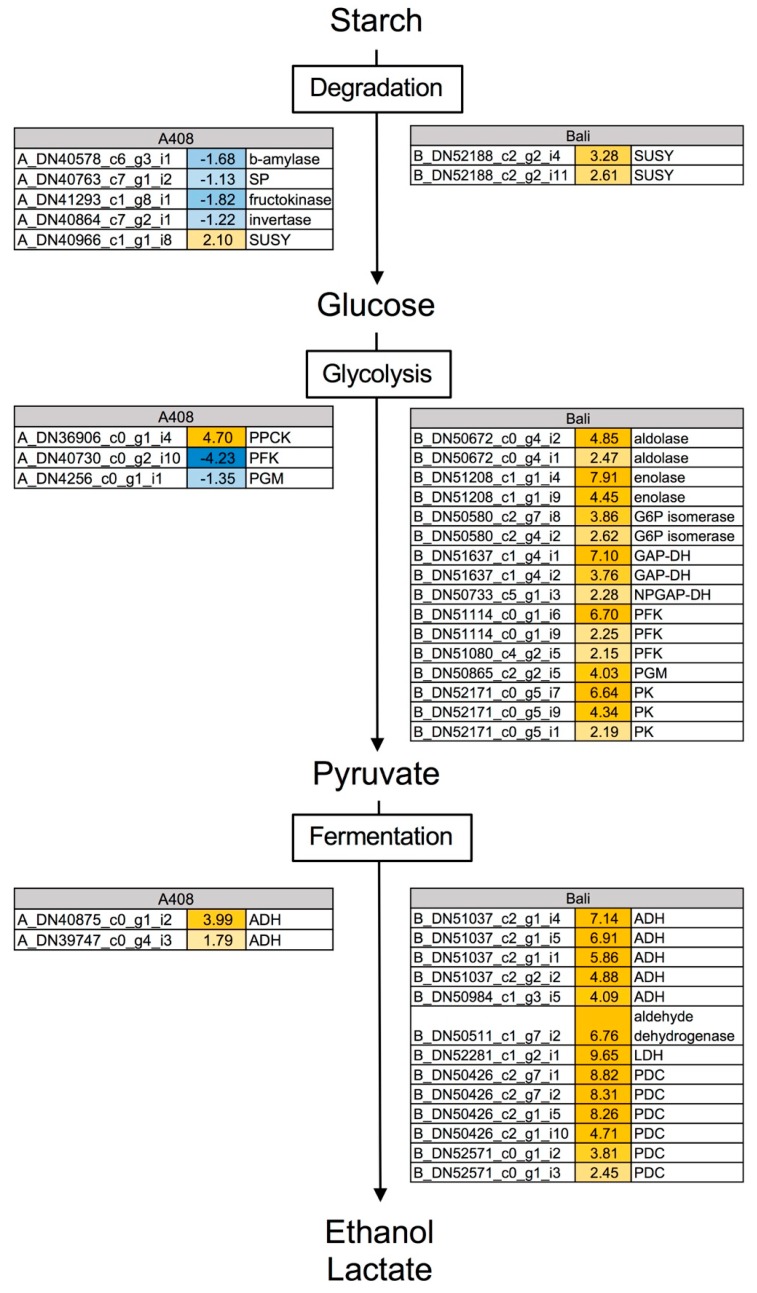
Waterlogging caused differential expression of major carbohydrate metabolism, glycolysis, and fermentative genes in roots of “A408” and “Bali”. The number indicates log_2_ fold changes. Blue indicates down-regulation. Yellow indicates up-regulation. Data can be found in Table S3.

Since WS creates a low oxygen environment that could promote glycolysis and fermentation and the GO enrichment and ORA analyses suggested differential expressions of glycolysis and fermentative genes in both varieties, we then examined changes in the expression of major carbohydrate metabolic, glycolysis and fermentative genes (Figure 6; Table S3). Starch degradation genes including *beta-amylase* (*A_DN40578_c6_g3_i1*), *starch phosphorylase* (*SP*; *A_DN40764_c7_g1_i2*), *fructokinase* (*A_DN41293_c1_g8_i1*), and *invertase* (*A_DN40864_C7_g2_i1*) were downregulated in “A408”. However, the expression of *sucrose synthase* (*SUSY*) was upregulated in both “A408” (*A_DN40966_c1_g1_i8*) and “Bali” (*B_DN52186_c2_g2_i4* and *i11*). Several genes encoded for glycolysis enzymes were strongly upregulated in “Bali”, including *aldolase* (*B_DN50672_c0_g4_i1* and *i2*), *enolase* (*B_DN51208_c1_g1_i4* and *i9*), *glucose 6 phosphate* (*G6P*) *isomerase* (*B_DN50580_c2_g7_i2* and *i8*), *GAP-DH* (*B_DN51637_c1_g4_i1* and *i2*), *phosphofrucktokinases* (*PFKs*; *B_DN51114_c0_g1_i6* and *i9* and *B_DN51080_c4_g2_i5*), *phosphoglycerate mutase* (*PGM*; *B_DN50865_c2_g2_i5*), and *pyruvate kinases* (*PKs*; *B_DN52171_c0_g5_i1*, *i7* and *i9*).

On the other hand, the analysis of “A408” DEGs revealed only one glycolysis gene, *phospho-enol-pyruvate carboxylase kinase* (*PPCK*; *A_DN36906_c0_g1_i4*), which was induced by WS. Interestingly, *PFK* (*A_DN40730_c0_g2_i10*), encoded for one of the most important regulatory enzymes of glycolysis, was strongly downregulated under WS in “A408”. Several fermentative genes were strongly upregulated in “Bali”, particularly *alcohol dehydrogenases* (*ADH*; *B_DN51037_c2_g1_i1*, *i2*, *i4*, and *i5* and *B_DN50984_c1_g3_i5*), *aldehyde dehydrogenase* (*B_DN50511_c1_g7_i2*), *lactate dehydrogenase* (*LDH*; *B_DN52281_c1_g2_i1*) and *pyruvate decarboxylase* (*PDC*; *B_DN50426_c2_g1_i5* and *i10*, *B_DN50426_c2_g7_i1* and *i2*, and *B_DN52571_c0_g1_i2* and *i3*). In contrast, only two genes encoding for *ADH* (*A_DN39747_c0_g4_i3* and *A_DN40875_c0_g1_i2*) were upregulated in “A408”. Our results demonstrated that genes involved in starch degradation, glycolysis, and fermentation are differentially expressed at a significantly higher level under WS in “Bali” than in “A408”, suggesting that “A408” could have a slower glycolytic process and a better ability to maintain carbohydrate reserves than “Bali”.

Analysis of total soluble carbohydrates in the roots of both varieties confirmed that WS resulted in a greater reduction of the total soluble carbohydrate in “Bali” than that of “A408” (Figure S1). Our results correlate with a study of wild relatives of *Arabidopsis* in the genus *Rorippa*, showing that starch degradation, glycolysis, and fermentative genes are more strongly induced in the less flooding-tolerant *R. amphibiathan* than in *R. sylvestris* [36], thus suggesting that the management of carbohydrate reserves may be necessary for the survival of plants experiencing an energy crisis from low oxygen conditions.

### 2.5. Waterlogging Resulted in Stronger Induction of Ethylene Synthesis, Perception and Responsive Genes in “Bali” than in “A408”

**Figure 7:**
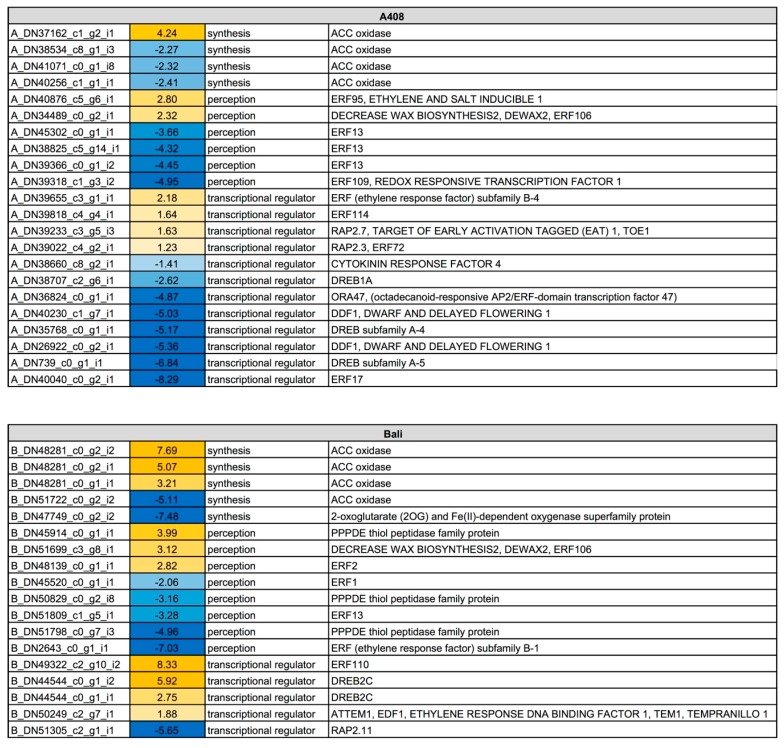
Differential expression pattern of ethylene synthesis, perception and transcriptional regulator genes in roots of “A408” and “Bali” subjected to WS. The number indicates log_2_ fold changes. Blue indicates down-regulation. Yellow indicates up-regulation. Data can be found in Table S3.

During soil waterlogging, ethylene acts as a primary signal controlling morphological and metabolic adjustments in plant roots. Therefore, we examined the changes in gene expression of ethylene synthesis, perception, and responsive transcriptional regulator genes. In both varieties, changes in the expression of ethylene synthesis, perception, and responsive transcriptional regulatory genes were observed (Figure 7; Table S3). In “A408” one aminocyclopropane carboxylate oxidase gene (*ACC oxidase*; *A_DN37162_c1_g2_i3*), a key-enzymatic gene controlling ethylene synthesis, was upregulated and three were downregulated (*A_DN38534_c8_g1_i3*, *A_DN41071_c0_g1_i1*, and *A_DN40256_c1_g1_i1*). On the contrary, two ACC oxidase genes (*B_DN48281_c0_g1_i1* and *B_DN48281_c0_g2_i1* and *i2*) were strongly upregulated, and one was downregulated (*B_DN51722_c0_g2_i2*) in “Bali”. For ethylene signaling and perception, *ERF95* (*A_DN40876_c5_g6_i1*) and *ERF106* (*A_DN34489_c0_g2_i1*) were upregulated in “A408” and *ERF2* (*B_DN48139_c0_g1_i1*) and *ERF106* (*B_DN51699_c3_g8_i1*) were upregulated in “Bali”. The down-regulation of *ERF13* genes was observed in both varieties (“A408”; *A_DN45302_c0_g1_i1*, *A_DN38825_c5_g14_i1*, and *A_DN39366_c0_g1_i2* and “Bali”; *B_DN51809_c1_g5_i1*). Interestingly, the down-regulation of *ERF109* (*A_DN39318_c1_g3_i2*), a redox responsive transcription factor 1, was observed only in “A408”. In *Arabidopsis*, *ERF109* is highly responsive to jasmonic acid and functions in the regulation of lateral root formation by mediating cross-talk between jasmonic acid signaling and auxin biosynthesis [37]. The expression of other ethylene-responsive transcription factor genes was generally strongly induced in “Bali”, including *ERF110* (*B_DN49322_c2_g10_i2*), *DREB2C* (*B_DN44544_c1_g1_i1* and *i2*) and *Ethylene Response DNA binding factor 1* (*B_EDF1*; *DN50249_c2_g7_i1*). In “A408”, *ERF subfamily B4* (*A_DN39655_c3_g1_i1*), *ERF114* (*A_DN39818_c4_g4_i1*), *RAP2.7* (*A_DN39022_c4_g2_i1*) and *RAP2.3* (*A_DN39022_c4_g2_i1*) were upregulated by WS. On the other hand, *DREB* transcription factors (*A_DN38707_c2_g6_i1*, *A_DN35768_c0_g1_i1*, and *A_DN739_c0_g1_i1*) were downregulated in “A408”.

The stronger up-regulation of *ACC oxidase* could result in higher ethylene production in “Bali” than in “A408”. Ethylene has an important role during lateral root initiation as treatment of ethylene reduces lateral root initiation in *Arabidopsis* seedlings [38]. Moreover, *Arabidopsis* mutants with enhanced ethylene synthesis or perception decreased lateral root formation, while ethylene-insensitive mutants increased the number of lateral roots [38]. Furthermore, Muday et al. [39] discussed the antagonistic roles of auxin and ethylene in controlling lateral root formation, in which the control of lateral root development by ethylene involves changes in auxin transport and accumulation patterns [40].

### 2.6. Auxin Metabolism and Auxin-Regulated Transcription Factor Genes were Predominantly Induced in the Roots of Waterlogging-Tolerant Zombi Pea

**Figure 8:**
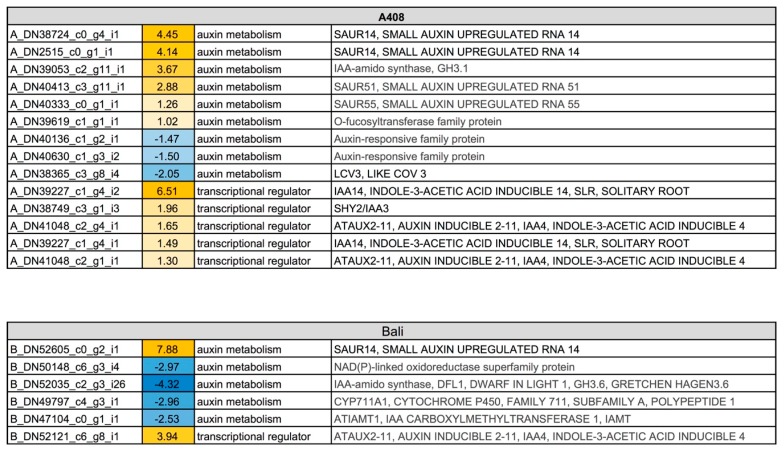
Differential expression pattern of auxin metabolism and transcriptional regulator genes in roots of “A408” and “Bali” subjected to WS. The number indicates log_2_ fold changes. Blue indicates down-regulation. Yellow indicates up-regulation. Data can be found in Table S3.

Auxin participates in root growth and the regulation of lateral root development. Our phenotypic data demonstrated that WS resulted in adaptive changes of zombi pea root phenotypes (Figure 2). GO enrichment analysis also suggests the down-regulation of IAA carboxyl methyltransferase activity in WS “Bali” roots (Figure 4). Recently, IAA methylation was proposed to function in maintaining auxin homeostasis by regulating the polar auxin transportation [41]. Moreover, the *AUX/IAA* family is overrepresented in the upregulated DEGs of “A408” based on Fisher’s exact test for over-representation analysis (Figure 5; Table S4). Therefore, we examined the changes in the expression of auxin metabolism and auxin-responsive transcription factor genes. Under WS, genes involved in auxin metabolism and auxin-responsive transcription factor genes were differentially regulated in both varieties (Figure 8; Table S3). However, four *Small Auxin Upregulated RNAs* (*SAURs*) were induced in “A408” (*A_DN2515_c0_g1_i1*, *A_DN38724_c0_g4_i1*, *A_DN40333_c0_g1_i1*, and *A_DN40413_c3_g11_i1*). In contrast, only one *SAUR* (*B_DN52605_c0_g2_i1*) was induced in “Bali”. Evidently, *SAURs* can regulate auxin-induced acid growth as defined by the loosening of cell walls at low pH which promotes cell wall extensibility and rapid cell elongation [42]. Our results demonstrate that WS in “A408” can upregulate a SAUR gene, *A_DN40413_c3_g11_i1*. The best BLAST hit of *A_DN40413_c3_g11_i1* protein is the *Arabidopsis* SAUR51 (*AT1G75580*; Table S3). Previous studies in *Arabidopsis* demonstrated that *SAUR51* is an auxin-inducible gene [43] which is highly expressed in root primordia [44], which suggests it may function in lateral root growth under WS.

Additionally, we observed the up-regulation of two key regulators in auxin-regulated lateral root development, *SHORT HYPOCOTYL 2/SUPPRESSOR OF HY 2 (SHY2)/Indole-3-acetic acid-inducible (IAA) 3* (*A_DN38749_c3_g4_i1*) and *IAA14* (*A_DN39227_c1_g4_i1* and *i2*) only in the DEGs of “A408”. SHY2/IAA3 and IAA14 are auxin-inducible transcriptional repressors that regulate auxin-mediated gene expression by controlling the activity of auxin response factors (ARFs) by protein-protein interaction [45,46,47]. Goh et al. [48] proposed that multiple *Aux/IAA–ARF* modules cooperatively regulate the developmental steps during lateral root formation. Therefore, we speculated that *SHY2/IAA3* and *IAA14* could specifically regulate zombi pea lateral root formation under WS. An in-depth analysis of the WS-induced *SAURs* and *Aux/IAAs* is required to further provide candidate genes for improving waterlogging tolerance in *Vigna* crops.

### 2.7. Differential Expression of Abscisic Acid and Gibberellic Acid Metabolic Genes

Abscisic acid (ABA) and gibberellic acid (GA) play antagonistic roles to control plant development and response to environmental stresses. GO enrichment analysis also suggests the down-regulation of farnesyltranstransferase activity and gibberellin 3-beta-dioxygenase activity in WS “Bali” roots (Figure 4). Additionally, ORA identified overrepresentation of GA metabolism genes in the downregulated DEGs of WS “Bali” roots (Figure 5). The biosynthesis of ABA and GA both derives from the isoprenoid pathway. Cutler et al. [49] demonstrated that *farnesyl transferase* is a key regulator of ABA signal transduction in *Arabidopsis*. Interestingly, the down-regulation of *farnesyl transferase* increases the ABA response and drought tolerance in *Brassica napa* [50]. In this study, the down-regulation of *genenylgeranyl pyrophosphate synthase 1* (*GGPS1*; *B_DN993_c0_g1_i1*) was observed in WS “Bali” roots (Table S3). *Arabidopsis* GGPS1 (encoded by *At4g36810*) has farnesyl transferase activity and functions as a key enzyme in the chloroplast isoprenoid biosynthetic pathway. GGPS1 catalyzes the formation of geranylgeranyl diphosphate, a precursor molecule of carotenoids, ABA, and GA [51]. Moreover, the down-regulation of *GA 3-oxidase 1* (*GA3OX1*: *B_DN46875_c0_g1_i1*) was also found in WS “Bali” roots (Table S3). *Arabidopsis GA3OX1* is involved in the production of bioactive GA, and plays an essential role in the regulation of root growth [52]. Altogether, these results suggest the modulation of ABA and GA level could play a role in the regulation of waterlogging tolerance in zombi pea.

### 2.8. Differential Expression of Transport Genes

**Figure 9:**
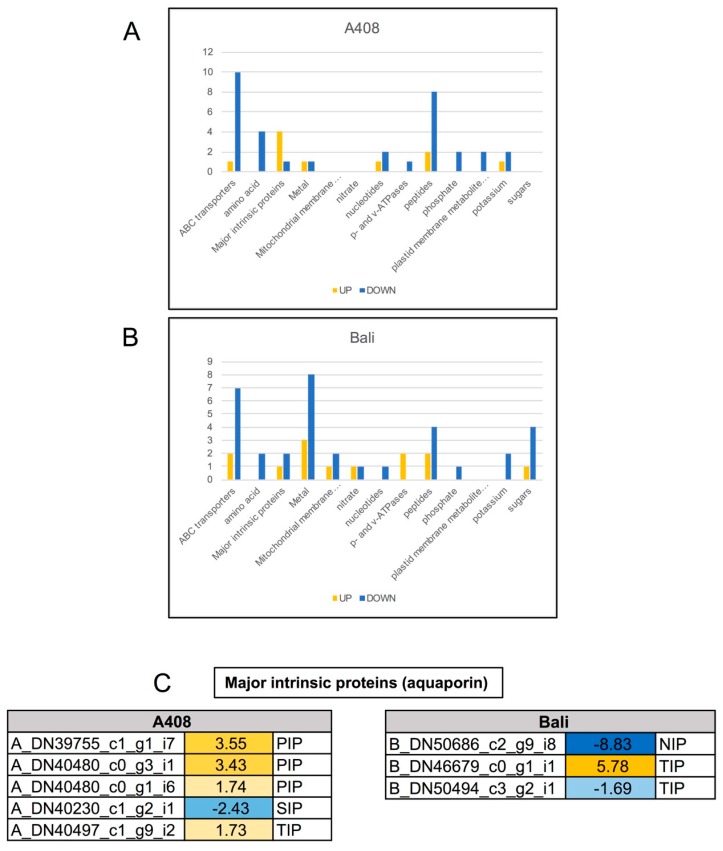
Differential expression of transport genes in roots of “A408” and “Bali” subjected to WS. Graphical representation of WS-regulated transport genes based on their assigned protein families. “Up” and “Down” represent up-regulation and down-regulation in this analysis. (**A**) “A408”. (**B**) “Bali”. (**C**) Expression patterns of major intrinsic protein (aquaporin) genes in roots of “A408” and “Bali” under WS. The number indicates log_2_ fold changes. Blue indicates down-regulation. Yellow indicates up-regulation. Data can be found in Table S3.

In general, most of the transporter gene families were downregulated in response to WS in both varieties (Figure 9A,B), except for a family of major intrinsic protein (aquaporin) genes which were largely induced under WS in “A408” (Figure 9A,B). The aquaporin has an important role in the regulation of plant water uptake, water loss, and hydraulic conductivity [53]. Among these aquaporin genes, three *plasma membrane intrinsic proteins* (*PIPs*) (*A_DN39755_c1_g1_i7*, *A_DN40480_c0_g1_i6*, and *A_DN40480_c0_g3_i1*) were specifically induced in “A408” (Figure 9C; Table S3). The best BLAST hit of *A_DN39755_c1_g1_i7* protein is the *Arabidopsis* PIP2;7 (*AT4G35100*; Table S3). Functional analysis of the *Arabidopsis PIP2;7* revealed that it is highly expressed in root elongating cells, and is most likely involved in cell elongation processes where the regulation of water movement is crucial [54]. Taken together, these results suggest the upregulation of aquaporin genes may contribute to waterlogging tolerance in the zombi pea.

### 2.9. Differential Expression of Plant Cell Wall-Related Genes

**Figure 10:**
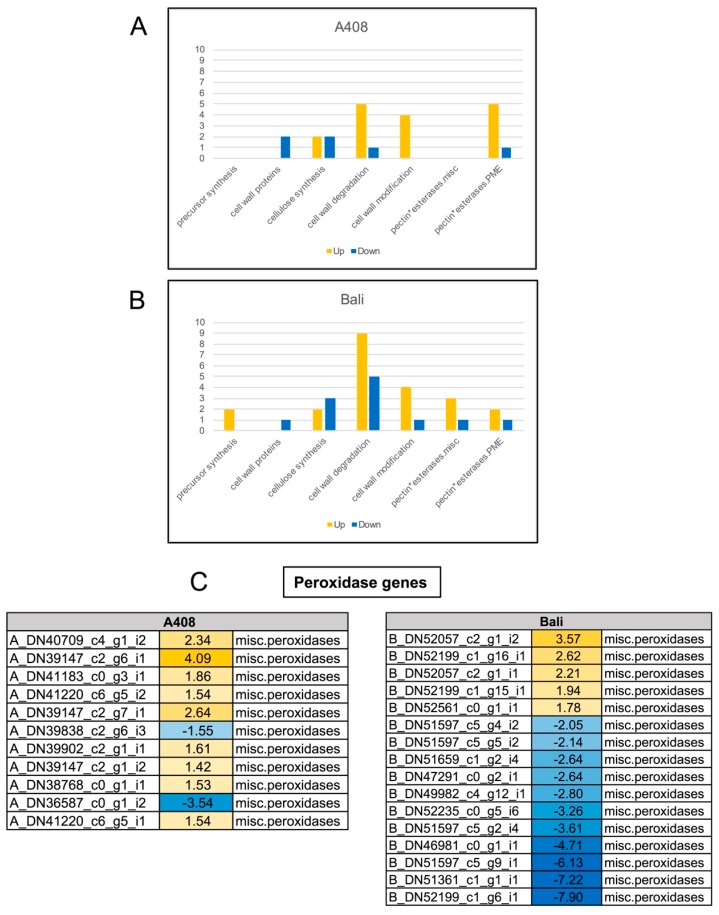
Differential expression of cell wall-related and peroxidase genes in roots of “A408” and “Bali” subjected to WS. Graphical representation of WS-regulated cell wall-related genes based on their assigned protein families. “Up” and “Down” represent up-regulation and down-regulation in this analysis. (**A**) “A408”. (**B**) “Bali”. (**C**) Expression patterns of peroxidase genes in roots of “A408” and “Bali” under WS. The number indicates log_2_ fold changes. Blue indicates down-regulation. Yellow indicates up-regulation. Data can be found in Table S3.

Since the aerenchyma formation was observed in WS roots and hypocotyl of “A408” (Figure 3A,B), we sought to determine the change in expression of cell wall-related genes upon WS. Our results demonstrated that cell wall-related genes were overrepresented in the upregulated DEGs of “A408” (Figure 5; Table S4). Genes involved in cell wall modification were overrepresented in the upregulated DEGs of both “A408” and “Bali” (Table S4). However, the group of pectin methylesterase genes, including both *pectin methylesterases* and *pectin methylesterase inhibitors*, were specifically overrepresented in the upregulated DEGs of “A408” (Figure 10A,B; Table S4). Pectin is a structurally compact polysaccharide that is a constituent of plant’s primary cell wall. Pectin plays a key role in plant growth, cell expansion, and response to stress [55]. Pectin methylesterases and pectin methylesterase inhibitors are enzymes involved in shoot apical meristem development and root tip elongation through plant hormone pathways including auxin [56,57]. Our results suggest that the modification of the plant cell walls by pectin methylesterases and pectin methylesterase inhibitors could have a role in the waterlogging tolerance of zombi pea. In support of this, *Glyma.03.g029400*, a soybean root-specific *pectin methylesterase inhibitor*, has been proposed as the likely underlying gene of a major QTL for waterlogging tolerance, *qWT_Gm03* [17].

Cell wall-associated peroxidases are enzymes that use hydrogen peroxide and/or superoxide anions as substrates to catalyze a production of hydroxyl radicals. The production of hydroxyl radicals can cause an increase in cell wall loosening during auxin-mediated cell wall extension. Here, we observed the over-representation of peroxidase genes in the upregulated DEGs of “A408” (Figure 5; Table S4). In the DEGs of “A408”, nine out of 11 peroxidase DEGs were upregulated under WS (Figure 10C). In contrast, only five out of 16 peroxidase DEGs were upregulated in the DEGs of “Bali” (Figure 10C). Interestingly, the expression of *A_DN39902_c2_g1_i1* and *A_DN40709_c4_g1_i2* was upregulated only in the DEGs of “A408” (Figure 10C). The best blast hit of these two transcripts is the *Arabidopsis* cell wall loosening peroxidase 53 (Prx53: AT5G06720; Table S3) [58]. The results suggest that these genes might play some role in waterlogging response.

### 2.10. Validation of Transcriptome Data by Quantitative Real-Time PCR

To validate the transcriptome results, for each variety we selected six DEGs and one non-DEG based on their function and expression level for quantitative real-time PCR analysis (qRT-PCR). For “A408”, of the six DEGs, five genes were upregulated including *pectin lyase-like superfamily protein* (*A_DN38641_c0_g1_i3*), *SUSY* (*A_DN40966_c1_g1_i8*), *ADH* (*A_DN39747_c0_g4_i3*), *aquaporin tonoplast intrinsic protein* (*TIP*; *A_DN40497_c1_g9_i2*), and *IAA14* (*A_DN39227_c1_g4_i1*) and one DEGs, *WRKY transcription factor* (*A_DN40719_c0_g11_i2*) was downregulated (Figure S2A). For “Bali”, of the six DEGs, four genes were upregulated including *ADH* (*B_DN50984_c1_g3_i5*), *GAP-DH* (*B_DN51637_c1_g4_i2*), *glucose-6-phosphate isomerase* (*B_DN50580_c2_g4_i2*), aldolase (*B_DN50672_c0_g4_i1*) and two DEGs, *TIP* (*B_DN50494_c3_g2_i1*) and *auxin-induced protein PCNT115* (*B_DN50148_c6_g3_i4*) were downregulated (Figure S2B). The expression of a non-DEG, *ATP synthase subunit beta* (“A408”; *A_DN39747_c0_g13_i1* and “Bali”; *B_DN50009_c1_g1_i3*), was used as a reference for the relative gene expression calculation. Our qRT-PCR results demonstrate the reliability of the RNA-seq data.

## 3. Material and Methods

### 3.1. Plant Material and Stress Treatment

*Vigna vexillata* seeds (JP235863 (“Bali”) and AusTRCF320047 (“A408”) varieties) were germinated in soil and grown outdoors between April and June of 2016 and 2017 at Kasetsart University, Bang Khen campus. Fifteen-day-old, five-leaf-stage plants were used in the WS treatment. In brief, plant pots were placed in plastic containers filled with tap water. The level of water was set at 3 cm above the soil. Waterlogging stress began at midday and continued for 24 h. For the control, non-treated plants were placed in a container with no water. For each sample, the root tissue of five plants was harvested at the end of the treatment; it was immediately placed in liquid nitrogen, ground into a fine powder, and kept at −80 °C. For long term WS, plants were subjected to WS for up to 10 days.

### 3.2. Analysis of Leaf Chlorophyll Content

Adhering to the method described by Juntawong et al. [2], chlorophyll content was measured using the atLEAF+ chlorophyll meter (FT Green LLC, Wilmington, DE). The youngest fully-expanded leaves were measured three times at 10.00 am and the averages were used in subsequent analysis. Twelve plants were analyzed for each time point. The total chlorophyll content of the leaves was obtained by converting the atLEAF+ values in SPAD units and the total chlorophyll contents using an online web tool: http://www.atleaf.com/SPAD.aspx.

### 3.3. Root Anatomical and Morphological Analysis

For analyses of root morphology, underground roots were collected and photographed after seven days of WS. Roots of NS plants grown side by side were used as controls. For the anatomical study, taproots and hypocotyls were immediately fixed in formaldehyde-acetic acid-alcohol (FAA) solution. Permanent slides for microscopic observation were prepared by standard microtechnique procedures [59]. The embedded samples were sectioned at 10–15 micrometer thickness using a rotary microtome (Leica RM2165; Leica Biosystems, Germany) and stained with Safranin and Fast Green. The samples were observed under a bright-field microscope (Axioskop 2 Plus; Zeiss, Germany) equipped with a digital camera (AxioCam MRc; Zeiss, Germany)

### 3.4. Analysis of Total Soluble Carbohydrate Content

One hundred milligrams of frozen root tissue was used to quantify the total soluble carbohydrate content using a method described by Juntawong et al. [2]. In brief, soluble carbohydrates were extracted, hydrolyzed by adding 5 mL of 2.5 N HCl, and incubated in a boiling water bath for 3 h. The addition of 0.75 g of Na_2_CO_3_ was performed to neutralize the extract. The anthrone method was used to determine total carbohydrate content relative to a standard series of glucose. In brief, the extract (300 μL) and distilled water (700 μL) were mixed with 4 mL of 0.14% (*w*/*v*) anthrone solution in 95% H_2_SO_4_; it was then incubated in a boiling water bath for 8 min, and rapidly cooled on ice. The absorbance was quantified at 630 nm.

### 3.5. RNA Extraction, Library Preparation, and Sequencing

Total RNA was extracted with TRIzol reagent (Invitrogen), according to the manufacturer’s protocol. Total RNA samples were subjected to DNase treatment and RNA cleanup using an RNA-mini kit (Qiagen). Two replicates of total RNA samples were used for transcriptome analysis according to the ENCODE recommended RNA-seq standards (https://genome.ucsc.edu/ENCODE/protocols/dataStandards/ENCODE_RNAseq_Standards_V1.0.pdf). The integrity of the RNA samples (RIN) was evaluated on an RNA 6000 Nano LapChiprun on Agilent2100 Bioanalyzer (Agilent Technologies, Germany). Samples with a RIN > 7 were used in RNA-seq library preparation. One μg of total RNAs were used to generate a sequencing library using a NEBNext^®^ Ultra™ RNA Library Prep Kit for Illumina^®^ following the manufacturer’s instructions. The mRNA fragmentation and priming were performed using NEBNext First Strand Synthesis Reaction Buffer and NEBNext Random Primers. First-strand cDNA was synthesized using ProtoScript II Reverse Transcriptase and the second-strand cDNA was synthesized using Second Strand Synthesis Enzyme Mix. The purified (via AxyPrep Mag PCR Clean-up (Axygen)) double-stranded cDNA was then treated with End Prep Enzyme Mix to repair both ends and added a dA-tailing in one reaction, followed by a T-A ligation to add adaptors to both ends. Size selection of Adaptor-ligated DNA was then performed using AxyPrep Mag PCR Clean-up (Axygen) and fragments of ~360 bp (with the approximate insert size of 300 bp) were recovered. Each sample was then amplified by PCR for 11 cycles using P5 and P7 primers, with both primers carrying sequences that could anneal with flow cell to perform bridge PCR and P7 primer carrying a six-base index allowing for multiplexing. The PCR products were cleaned using AxyPrep Mag PCR Clean-up (Axygen), validated using an Agilent 2100 Bioanalyzer (Agilent Technologies, Palo Alto, CA, USA), and quantified using Qubit 2.0 Fluorometer (Invitrogen, Carlsbad, CA, USA). Then libraries with different indices were multiplexed and loaded on an Illumina HiSeq 4000 instrument according to the manufacturer’s instructions (Illumina, San Diego, CA, USA). Sequencing was carried out using a 2 × 150 bp paired-end (PE) configuration; image analysis and base calling were conducted by the HiSeq Control Software (HCS) + RTA 2.7 (Illumina) on the HiSeq 4000 instrument. The raw read files were deposited in the NCBI SRA database under the accession numbers SRR9214917- SRR9214924. Quality control filtering and 3′ end trimming were analyzed using the FASTX-toolkit (http://hannonlab.cshl.edu/fastx_toolkit/index.html) and Trimmomatic software [60], respectively.

### 3.6. De Novo Assembly and Annotation

The transcriptome was assembled using the Trinity software (https://github.com/trinityrnaseq/trinityrnaseq) [61]. The assembly was performed using a k-mer value of 25 with default parameters. The de novo transcriptome assembled files can be found in Files S1 and S2. The protein sequences derived from the assembled transcriptomes were further annotated using BLASTP to plant UniprotPK database with an E value threshold of 1e−10 using AgBase (http://agbase.arizona.edu) and the Mercator annotation pipeline with a blast cut-off score of 80 (https://plabipd.de/portal/mercator-sequence-annotation). The annotation information can be found in Table S2.

### 3.7. Differential Gene Expression Analysis

Differential gene expression analysis was performed according to Sirikhachornkit et al. [62]. The FASTQ files were aligned to the reference transcriptome using Bowtie2 software (http://bowtie-bio.sourceforge.net/bowtie2/index.shtml). A binary format of sequence alignment files (BAM) was generated and used to create read count tables using the HTseq python library (https://htseq.readthedocs.io/). Differentially-expressed genes were calculated using the edgeR program (https://bioconductor.org/packages/release/bioc/html/edgeR.html) with an FDR cutoff of < 0.05.

Gene ontology enrichment analysis was performed in the R environment using the GOHyperGAll function [63]. Gene annotation files were generated using the AgBase webtool. Significant GO terms were filtered by an adjusted *p*-value of < 0.05.

For PAGEMAN analysis, the mapping file was generated from the protein sequences derived from the assembled transcriptomes using the Mercator pipeline. Over-representation analysis (ORA) was performed using the PAGEMAN [64] program with Fisher’s test and a cutoff value of two.

Homolog identification was performed using translated amino acid sequences (>100 amino acids) derived from the transcriptomes of “A408” and “Bali” and *A. thaliana* protein sequences (TAIR10) by OrthoVenn2 [65]. The homolog clusters and expression can be found in Table S3.

### 3.8. Quantitative Real-Time PCR

Three replicates of total RNA samples were used. Total RNAs were treated with DNase I (NEB, USA) to eliminate contaminated genomic DNA. One microgram of total RNAs were used to construct cDNA using MMuLv reverse transcriptase (Biotechrabbit, Germany) in a final volume of 20 μL. The cDNA was diluted five times. Quantitative-realtime PCR (qPCR) reaction was performed according to Sirikhachornkit et al. [62]. Further, qPCR was performed using QPCR Green Master Mix (Biotechrabbit, Germany) on a MasterCycler RealPlex ^4^ (Eppendorf, Germany). For each sample, the PCR reaction was performed in triplicate. Each reaction contained 1  μL of diluted cDNA, 0.5  μM of each primer, and 10  μL of QPCR Green Master Mix, giving a final volume of 20  μL. The PCR cycle was 95  °C for 2  min, followed by 45 cycles of 95  °C for 15  s and 60  °C for 30 s. Amplification specificity was validated by melt-curve analysis at the end of each PCR experiment. Relative gene expression was calculated using the 2 ^−∆∆CT^ method. The genes and primers used are shown in Table S5.

## 4. Conclusions

In this research, we aimed to discover the molecular mechanisms controlling waterlogging tolerance by constructing de novo transcriptomes and comparing the transcriptomes of two zombi pea varieties with contrasting waterlogging tolerance. Our results demonstrated that root plasticity could be an important determinant factor controlling waterlogging tolerance in zombi pea. Moreover, differential expressions of multiple genes encoding for energy production pathways, auxin-regulated lateral root initiation and formation, hormones, cell wall modification, membrane transporter, and peroxidase could contribute to waterlogging tolerance in zombi pea. Functional characterization of the WS-induced candidate genes is required to further identify candidate genes controlling waterlogging-tolerant traits. Additionally, recent studies demonstrated that differentially-regulated genes controlling for the traits of interest could be accurately identified using comparative transcriptome RNA-seq analysis of near-isogenic lines (NILs) [66,67]. Clearly, this method could help to narrow down the list of candidate genes responsible for waterlogging tolerance in zombi pea by removing genetic background effects. We expect that the basic knowledge obtained from this study will be used to help design further experiments focused on improving our understanding of the morphological and physiological responses to waterlogging that are important for molecular breeding of waterlogging-tolerant *Vigna* crops in the future.

## Supplementary Materials

The following are available online at https://www.mdpi.com/2223-7747/8/8/264/s1, File S1: “A408” *de novo* assembled transcriptome data. File S2: “Bali” *de novo* assembled transcriptome data. Figure S1: Total root carbohydrate content. (A) “A408”. (B) “Bali”. Data represent mean ± SE (*n* = 3). * *p* < 0.05, ** *p* < 0.01 (*t*-test). Figure S2: Quantitative real-time PCR validation of transcriptome data for selected genes. (A) “A408”. (B) “Bali”. Relative expression was normalized to the abundance of *ATP synthase subunit beta*. Data represent mean ± SE (*n* = 3). * *p* < 0.05, ** *p* < 0.01 (*t*-test). Table S1: Transcriptome statistics. Table S2: Transcriptome annotation. Table S3: Differentially-expressed transcripts. Table S4: Comparative transcriptome analysis. Table S5: List of genes and primers used for qRT-PCR.
