# Supplementary figures and images for: Comparative Transcriptome Analysis of Waterlogging-Sensitive and Tolerant Zombi Pea (Vigna vexillata) Reveals Energy Conservation and Root Plasticity Controlling Waterlogging Tolerance
Source: Plants (Basel). 2019 Aug 2;8(8):264. doi: 10.3390/plants8080264 (PMC6724125; doi:10.3390/plants8080264)

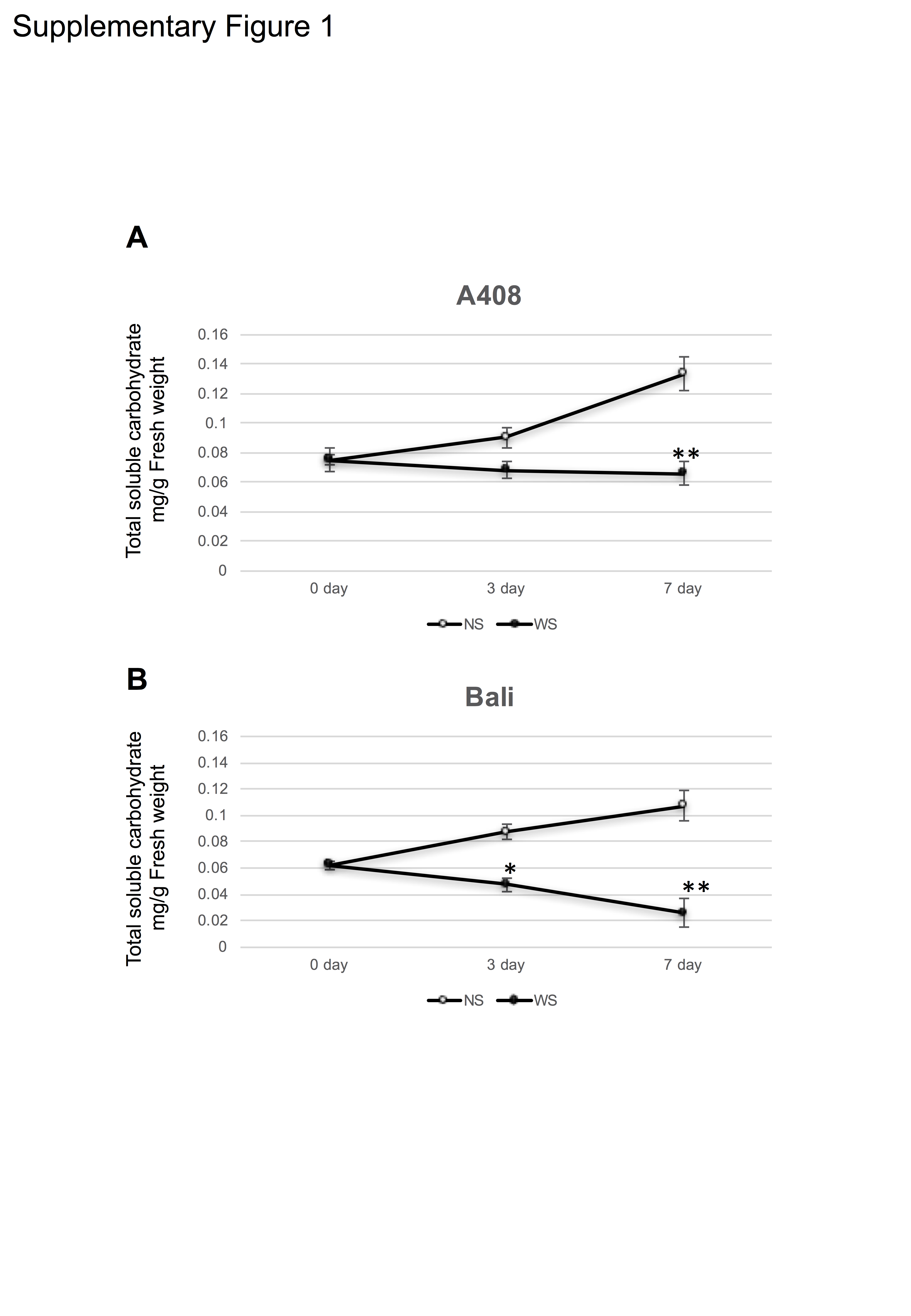

Supplement: Supplementary file 1 [file plants-08-00264-s001.zip › Supplementary material/Supplementary Figure_S1.jpg]

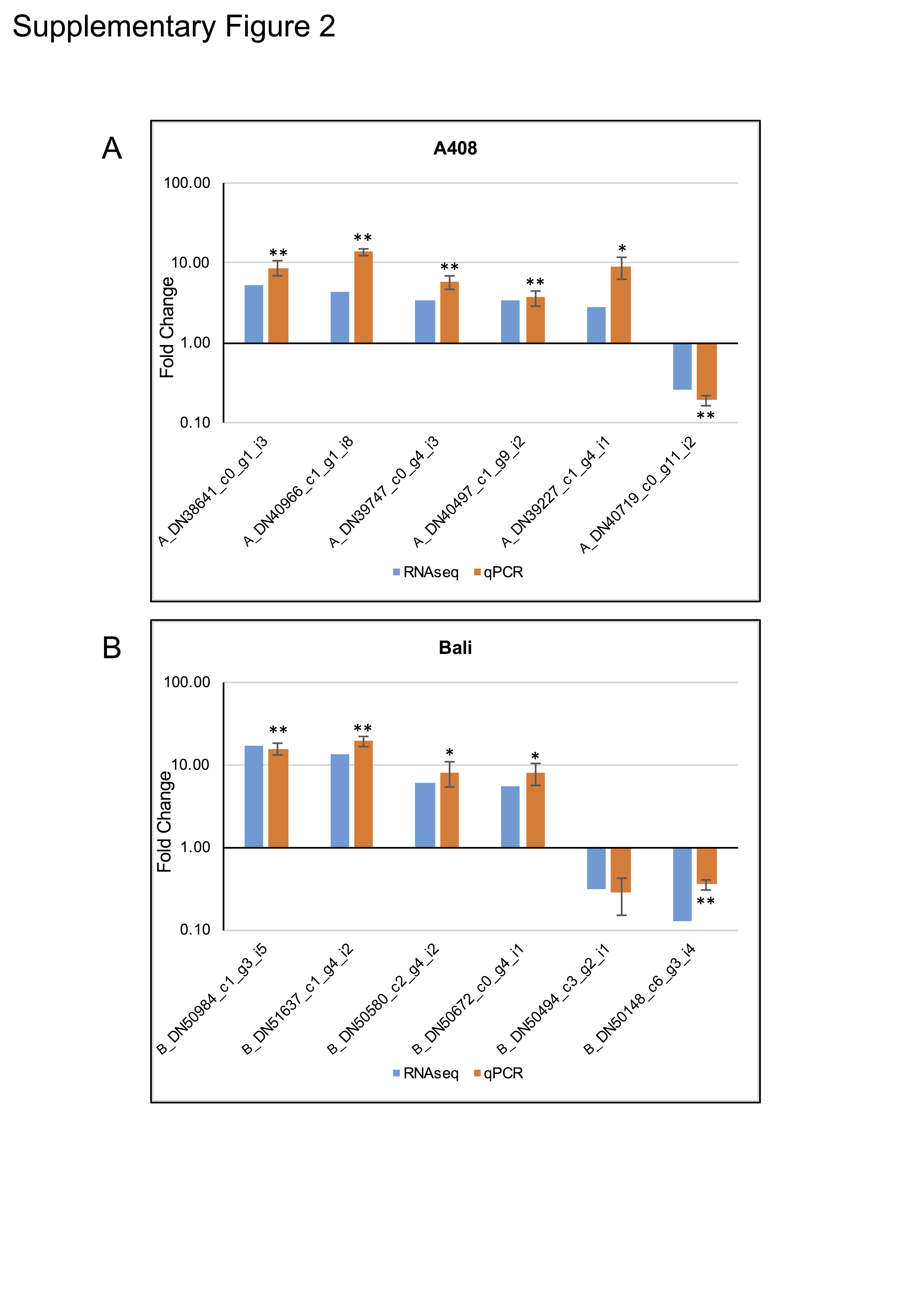

Supplement: Supplementary file 1 [file plants-08-00264-s001.zip › Supplementary material/Supplementary Figure_S2.jpg]
